# Regulatory role of the RstB‐RstA system in adhesion, biofilm production, motility, and hemolysis

**DOI:** 10.1002/mbo3.599

**Published:** 2018-03-23

**Authors:** Lixing Huang, Wei Xu, Yongquan Su, Lingmin Zhao, Qingpi Yan

**Affiliations:** ^1^ State Key Laboratory of Large Yellow Croaker Breeding Ningde Fujian China; ^2^ Fisheries College Key Laboratory of Healthy Mariculture for the East China Sea Ministry of Agriculture Jimei University Xiamen Fujian China; ^3^ Third Institute of Oceanography State Oceanic Administration Xiamen China; ^4^ College of Ocean & Earth Sciences Xiamen University Xiamen Fujian China

**Keywords:** adhesion, *rstA*, *rstB*, *Vibrio alginolyticus*

## Abstract

For infection, initial invasion of the host is of great importance, with adhesion playing a critical role. We previously demonstrated *rstA* and *rstB* are remarkably downregulated in *Vibrio alginolyticus* cultured under heavy metal and acidic stresses, with impaired adhesion, suggesting that *rstA* and *rstB* might be involved in adhesion regulation. The present study showed that *rstA* and *rstB* silencing resulted in impaired adhesion, biofilm production, motility, hemolysis, and virulence. Meanwhile, changes of temperature, starvation, and pH remarkably affected *rstA* and *rstB* expression. These findings indicated that (1) *rstA* and *rstB* are critical regulators of adhesion in *V*. *alginolyticus*; (2) *rstA* and *rstB* have remarkable effects on biofilm production, motility, hemolysis, and virulence in *V*. *alginolyticus*; (3) *rstA* and *rstB* modulate adhesion in response to environmental changes of temperature, pH, and starvation.

## 1 INTRODUCTION

As a widespread opportunistic pathogen (Wang et al., 2014), *Vibrio alginolyticus* can cause vibriosis in cultured fish such as *Pseudosciaena crocea*, leading to severe economic loss (Wang et al., 2015; Wu et al., 2014).

Attachment to the host surface is the initial and vital part of the bacterial infection process (Pan, Yang, & Zhang, 2014; Pizarro‐Cerda & Cossart, 2006). Abundant mucus covers the host surface, including the lining of the gut, skin, and gills. Therefore, the mucus is the initial place where host‐pathogen interactions occur (Benhamed, Guardiola, Mars, & Esteban, 2014). Nowadays, bacterial attachment to the host mucus attracts increasing attention (Bergstrom et al., 2010; Guo et al., 2017; Huang et al., 2017; Jørgensen et al., 2012; Liu et al., 2013, 2017; Taghavi et al., 2015). However, studies assessing the mechanisms of adhesion in *V. alginolyticus* remain limited.

Our laboratory has evaluated *V. alginolyticus* attachment to *P. crocea* mucus for years. *V. alginolyticus* adhesion is affected by environmental changes of temperature, pH, starvation, and salinity (Qiang, Qingpi, Shen, Zhixia, & Xiaoru, 2007). Treatments with pH5, Pb, Cu, and Hg were reported to reduce adhesion in *V. alginolyticus* by 56.58%, 39.26%, 37.41%, and 40.65%, respectively (Kong et al., 2015). Using RNA‐Seq, Kong et al. (2015) demonstrated that pH5, Pb, Cu, and Hg remarkably reduce *rstA* and *rstB* expression levels. Indeed, Cu, Pb, Hg, and low pH remarkably down‐regulated *rstA* (by 2.17‐, 3.02‐, 2.14‐, and 8.01‐fold, respectively) and *rstB* (by 2.52‐, 3.59‐, 2.00‐, and 7.76‐fold, respectively).

The *rstA* and *rstB* genes constitute a two‐component system, which is a signal transduction system activated in response to environmental changes (Yamamoto et al., 2005). Every two‐component system includes a response regulator (RR) and a sensor protein‐histidine kinase (HK). Through histidyl‐aspartyl phospho‐relay, HK and RR form a signal transduction pathway. RstB and RstA are HK and RR, respectively. The function of the RstB‐RstA system in bacterial virulence is not well characterized. Tran, Han, Shi, and Guo (2016) found that invasion, motility, pyrimidine metabolism, and iron acquisition in *Salmonella typhimurium* are controlled by the RstB‐RstA system. Menanteau‐Ledouble and Lawrence (2013) demonstrated that mutation of the RstB‐RstA system results in decreased adhesion and virulence in *Edwardsiella ictaluri*. Terceti et al. (2017) reported that RstB plays key roles in the hemolytic activity and pathogenicity of *Photobacterium damselae*. As *V. alginolyticus* adhesion is likewise impaired under stress, the RstB‐RstA system was hypothesized to participate in this process.

This study aimed to examine whether the RstB‐RstA system contributes to *V. alginolyticus* attachment to the host mucus, by assessing (1) the association of *rstA* and *rstB* with *V. alginolyticus* attachment, and (2) whether *rstA* and *rstB* modulate adhesion in response to environmental stimuli.

## 2 MATERIALS AND METHODS

### 2.1 Bacterial strains and culture conditions

*Vibrio alginolyticu* strain ND‐01 is a clinical isolate from naturally infected *P*. *crocea* (Kong et al., 2015). It was cultured at 28°C in Luria–Bertani (LB) broth supplemented with 2% NaCl, with shaking (220 rpm).

To assess the effects of temperature on *rstA* and *rstB* expression levels, *V. alginolyticus* was incubated overnight in LB at 4°C, 15°C, 28°C, 37°C, and 44°C, respectively. The bacteria were harvested, resuspended, and incubated for 30 min at their respective culture temperatures (Huang, Huang, et al., 2016), in triplicate. *V. alginolyticus* cultured at 4°C was used as a control group here, which means the expression levels of *rstA* and *rstB* at 15°C–44°C were compared to their counterparts at 4°C.

To assess the effects of pH on *rstA* and *rstB* expression levels, *V. alginolyticus* cells were cultured at pH5, pH6, pH7, pH8, and pH9, respectively, overnight at 28°C, and washed with PBS at the same pH used for culture, in triplicate (Yan, Chen, Ma, Zhuang, & Wang, 2007). *V. alginolyticus* cultured at pH5 was used as a control group here, which means the expression levels of *rstA* and *rstB* at pH6‐9 were compared to their counterparts at pH5.

To evaluate the effects of starvation on *rstA* and *rstB* expression levels, *V. alginolyticus* was incubated in PBS and adjusted to OD_600 nm_ = 0.3, and starved for 1, 3, 5, and 7 days, respectively, at 28°C. The plate counting method was used to quantify culturable cells (Huang, Huang, et al., 2016; Jiang et al., 2017; Lin et al., 2017). Three replicates were set up. *V. alginolyticus* starved for 1 day was used as a control group here, which means the expression levels of *rstA* and *rstB* in *V. alginolyticus* starved for 3–7 days were compared to their counterparts in *V. alginolyticus* starved for 1 day.

### 2.2 Stable gene silencing

The *rstA* and *rstB* genes were silenced with vectors containing short hairpin RNA (shRNA) sequences targeting the *rstA* and *rstB* coding regions as previously described (Huang et al., 2015). The shRNA sequences were obtained from Shanghai Generay Biotech Co., Ltd. (Shanghai, China) (Table 1). Annealed oligonucleotides were ligated into the Tc operon of *Bam*HI and *Sph*I double digested pACYC184 vector using T4 DNA ligase (TaKaRa, Japan) (Qin et al., 2014). Recombinant plasmids were transformed into *Escherichia coli* SM10 (Dongsheng, Guangzhou, China) by heat shock. Recombinant plasmids were then transferred from strain SM10 into *V. alginolyticus* by conjugation. The empty pACYC184 vector was used as a control. Chloramphenicol was employed to screen clones with stable silencing at a concentration of 34 μg/ml.

**Table 1:** Oligonucleotides used in producing shRNA for stable gene silencing

Target	shRNA sequence for stable gene silence
*rstA*	F:5’‐GATCCGTGGAAGACGATCCCAAATTATTCAAGAGATAATTTGGGATCGTCTTCCACTTTTTTGCATG‐3’ R:5’‐CAAAAAAGTGGAAGACGATCCCAAATTATCTCTTGAATAATTTGGGATCGTCTTCCACG‐3’
*rstB*	F:5’‐GATCCGCAGATAATGGAACTTCAACATTCAAGAGATGTTGAAGTTCCATTATCTGCTTTTTTGCATG‐3’ R:5’‐CAAAAAAGCAGATAATGGAACTTCAACATCTCTTGAATGTTGAAGTTCCATTATCTGCG‐3’

### 2.3 RNA extraction and reverse transcription

TRAzol (Dongsheng, Guangzhou, China) was used for total RNA extraction from *V. alginolyticus*. A Revert Aid Mu‐MLV cDNA synthesis kit (Dongsheng, Guangzhou, China) was employed to synthesize first‐strand cDNA from total RNA. These experiments were conducted according to the manufacturer's instructions.

### 2.4 Quantitative RT‐PCR (qRT‐PCR)

Quantitative RT‐PCR was carried out on a QuantStudio^™^ 6 Flex real‐time PCR system (ABI, USA) with the SYBR green I fluorescent dye (Dongsheng, Guangzhou, China). The mRNA expression levels were determined as previously described (Liu et al., 2017). Primers were designed with Primer Premier 5.0 (Table 2). The expression levels of *rstA* and *rstB* were normalized to that of 16S RNA. Relative Expression Software Tool (REST 2008.‐version 2) was used to assess the relative expression levels of *rstA* and *rstB* by qRT‐PCR (Pfaffl, Horgan, & Dempfle, 2002).

**Table 2:** Primers for qRT‐PCR

Gene	Primers for qRT‐PCR
*rstA*	F: 5’ GTGAATGCTACAAAGGCAAAGTG 3’ R: 5’ TGCGAGAACCCATAATAAATCG 3’
*rstB*	F: 5’ GGTATAGAAGAGCAGCATTGGC 3’ R: 5’ GTGAAGCAAGCACCACCAAG 3’
*16S RNA*	F: 5’ GGGGAGTACGGTCGCAAGAT 3’ R: 5’ CGCTGGCAAACAAGGATAAGG 3’

### 2.5 Mucus preparation

Based on a previously described method (Kong et al., 2015), skin mucus was collected from healthy *P. crocea* in Ningde, Fujian Province, China. Briefly, the fish was washed with sterile PBS. For skin mucus collection, the surface gel layer of the skin was scrapped with a plastic spatula. The collected mucus was homogenized in PBS, and centrifuged twice at 4°C for 30 min (20,000*g*) to remove particulate materials. Then, 0.45‐ and 0.22‐μm pore filters were successively used for filtration. The mucus sample was adjusted to a final concentration of 1 mg protein/ml as previously proposed (Bradford, 1976).

### 2.6 In vitro adhesion assay

According to Huang, Hu, et al. (2016), *V. alginolyticus* adhesion was analyzed. Briefly, 50 μl of mucus was spread onto a glass slide (22 × 22 mm) evenly, and fixed with methanol. Twenty minutes later, 1 ml of bacterial suspension (10^8^ CFU/ml) was gently applied to mucus coated glass slides, and incubated for 2 hr in a humidified chamber at 25°C. The slides were then washed with PBS (5 times) to remove nonadherent bacteria. The specimens were then fixed with 4% methanol for 30 min, followed by crystal violet staining for 3 min. Finally, microscopic observation (×1,000) was carried out, and the average number of adherent bacteria was determined. In each assay, 20 fields of view were randomly selected.

### 2.7 Soft agar plate motility assay

By the soft agar approach, the flagellar motility of *V. alginolyticus* was evaluated. Overnight cultured *V. alginolyticus* was diluted to OD_600_ = 0.03. Then, 1 μl of the suspension was gently dropped onto the center of LB agar plates, followed by incubation at 28°C. After 20 hr of culture, colony diameters were measured.

### 2.8 Biofilm assay

As previously described (Luo et al., 2016), the biofilm assay was carried out for *V. alginolyticus*. Overnight *V. alginolyticus* cultures were adjusted to OD_600 nm_ = 0.2. Then, 150 μl of LB was mixed with 50 μl of bacterial culture per well in 96‐well plates. After incubation at 28°C for 24 hr, sterile PBS was used for 3 washes. The samples were stained for 15 min with 200 μl of 1% crystal violet, rinsed with sterile PBS, and air dried. Finally, 200 μl acetic acid (33%) was used for solubilizing the stained biofilm, which was quantitated by measuring OD_590 nm_. The experiment was performed in triplicate.

### 2.9 Hemolysis assay

As previously described (Tsou & Zhu, 2010), hemolysis assays were performed. First, commercial rabbit blood (PingRui, Beijing, China) was rinsed three times with PBS. After incubation of 245 μl of culture supernatant with 5 μl of washed rabbit blood at 37°C for 1 hr with shaking (220 rpm), detection of released hemoglobin was carried out by measuring OD_540 nm_. The percentage of total hemolysis was determined by comparison with negative and positive control samples (100% lysis by 1% Triton X‐100). The experiment was performed in triplicate.

### 2.10 Artificial infection

*Epinephelus coioides* was used for artificial infection as previously described (Liu et al., 2017). Sixty healthy *E. coioides* were randomly divided into 3 groups. Each fish was intraperitoneally administered 0.1 ml of *V. alginolyticus* suspension (10^7^ CFU/ml) of wild type and silenced strains, respectively. Instead of *V. alginolyticus* suspension, sterile PBS was used in the negative control group. Mortality was observed every day for 10 days.

### 2.11 Data processing

Results were reported as mean ± standard deviation (*SD*). Statistical analysis was conducted with the SPSS 13.0 software (SPSS, Chicago, IL, USA). Differences were assessed by one‐way analysis of variance (ANOVA) followed by Dunnett's multiple comparison test. *p* < .05 was considered statistically significant.

## 3 RESULTS

### 3.1 Validation of RNA‐seq data

**Figure 1:**
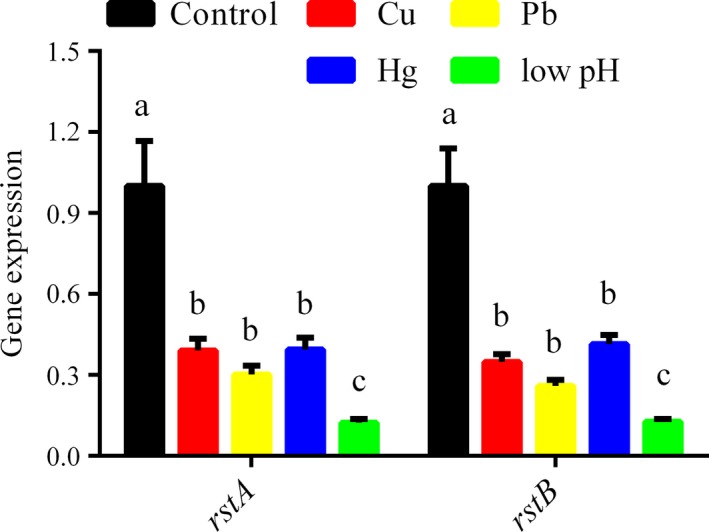
Quantitative RT‐PCR (qRT‐PCR) analysis of *rstA* and *rstB* gene expression levels after treatment with Cu, Pb, Hg, and low pH. Data are mean ± *SD* of 3 independent biological replicates. Means not sharing a common letter are significantly different (*p* < .05)

To validate RNA‐Seq data, qRT‐PCR was performed to assess *rstA* and *rstB* gene expression levels. The qRT‐PCR results were in accordance with RNA‐Seq data. Cu^2+^, Pb^2+^, Hg^2+^, and low pH remarkably down‐regulated *rstA* (by 2.56‐, 3.32‐, 2.54‐, and 8.13‐fold, respectively) and rst*B* (by 2.87‐, 3.85‐, 2.42‐, and 7.90‐fold, respectively) (Figure 1). These results supported the reliability of previous RNA*‐*Seq findings.

### 3.2 Effects of environmental changes on *rstA* and *rstB* expression levels

**Figure 2:**
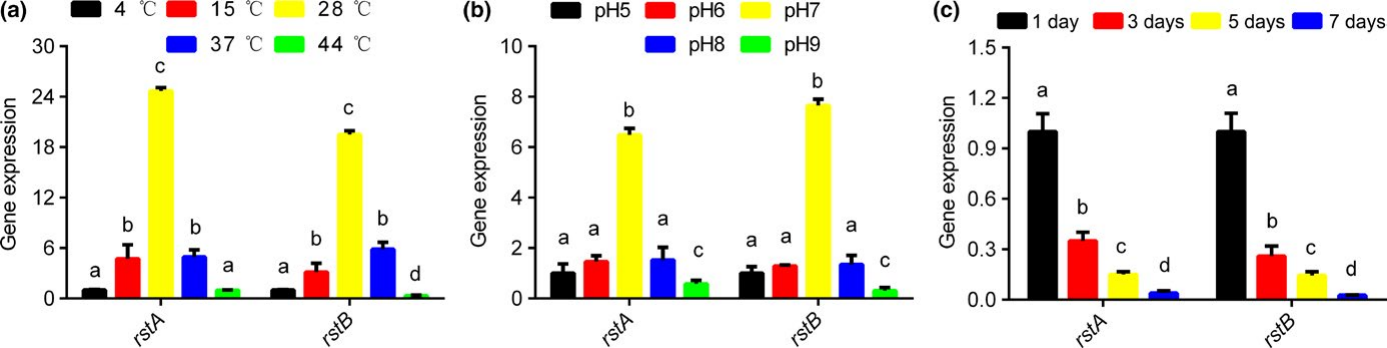
Quantitative RT‐PCR (qRT‐PCR) analysis of *rstA* and *rstB* gene expression levels in *V. alginolyticus* under different temperatures (a), pH values (b), and starvation times (c). *Vibrio alginolyticus* cultured at °C, pH5 and *V. alginolyticus* starved for 1 day were used as controls, respectively. Data are mean ± *SD* of 3 independent biological replicates. Means not sharing a common letter are significantly different (*p* < .05)

To evaluate *rstA and rstB* responses to temperature changes, their expression levels were detected at different temperatures (Figure 2A). The expression levels of both *rstA* and *rstB* showed an inverted U‐shaped trend. However, these genes showed highest levels at 28°C. These findings suggested that both *rstA and rstB* were sensitive to temperature changes.

To evaluate the responses of these genes to pH changes, their expression levels were assessed at different pH levels. As shown in Figure 2B, an inverted U‐shaped trend was also obtained. Highest expression levels were found at pH 7.0 for both *rstA* and *rstB*, indicating that they were sensitive to pH changes.

To evaluate the responses of these genes to starvation, their expression levels were assessed under starving conditions. Starvation resulted in remarkably decreased gene expression levels, in a time‐dependent manner (Figure 2C). These findings suggested that *rstA* and *rstB* were both sensitive to starvation.

### 3.3 Effects of *rstA* and *rstB* silencing on adhesion

**Figure 3:**
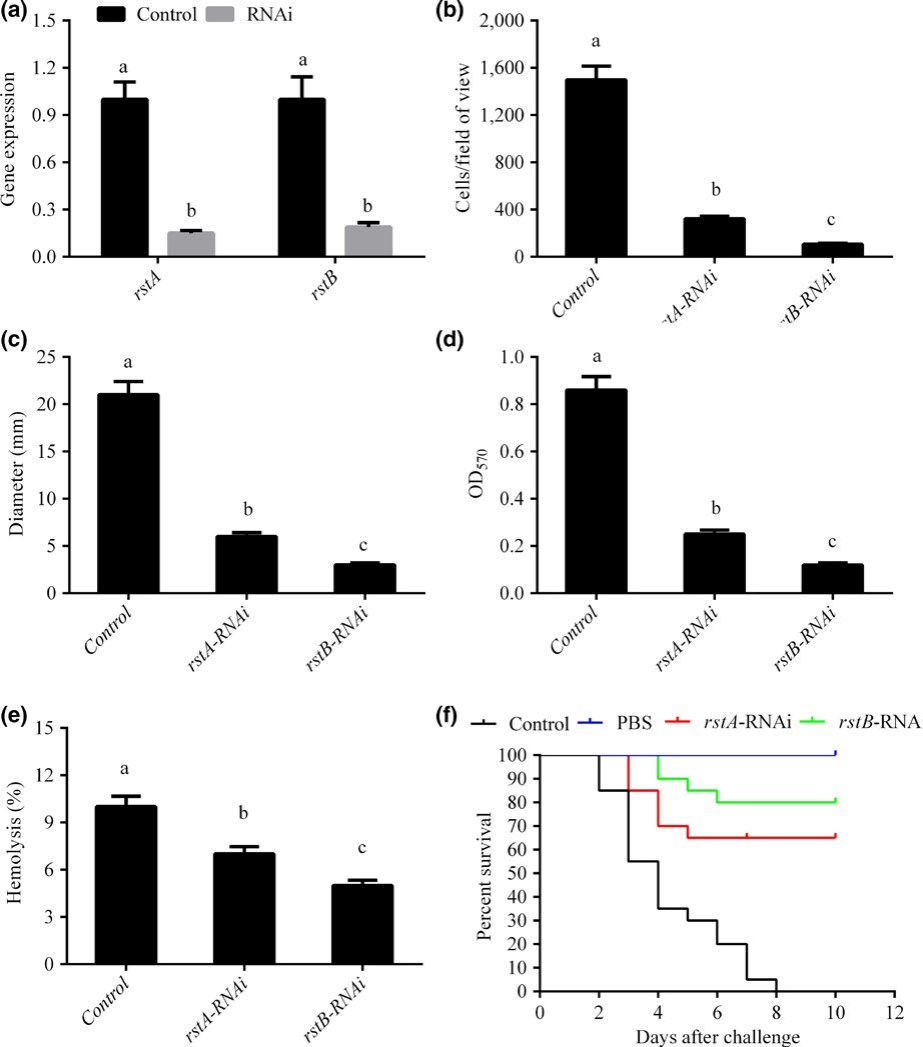
Effects of *rstA* and *rstB* silencing. (a) Quantitative RT‐PCR (qRT‐PCR) analysis of *rstA* and *rstB* gene expression levels after stable gene silencing compared with the control. Data are mean ± *SD* of 3 independent biological replicates. Means not sharing a common letter are significantly different (*p* < .05). (b) Adhesion on mucus after stable gene silencing in *V. alginolyticus*. Data are mean ± *SD* of 3 independent biological replicates. Means not sharing a common letter are significantly different (*p* < .05). (c) Motility behavior on soft agar plates after stable gene silencing in *V. alginolyticus*. Colony diameters for all strains are mean ± *SD* of three independent biological replicates. Means not sharing a common letter are significantly different (*p* < .05). (d) Biofilm formation after stable gene silencing in *V. alginolyticus*. OD_570_ values for stained biofilm are mean ± *SD* of three independent biological replicates. Means not sharing a common letter are significantly different (*p* < .05). (e) Stable gene silencing results in reduced hemolytic activity in *V. alginolyticus*. Hemolytic activities of *V. alginolyticus* are mean ± *SD* of three independent biological replicates. Means not sharing a common letter are significantly different (*p* <.05). (f) Percent survival of *Epinephelus coioides* administered wild‐type, and *rstA‐* RNAi and *rstB*‐RNAi strains, respectively, at 10 days postchallenge

As shown by qRT‐PCR, *rstA* and *rstB* were remarkably reduced in stably silenced clones, by 6.67‐ and 5.26‐fold, respectively (Figure 3A).

The adhesion capabilities of *rstA*‐ and *rstB*‐silenced clones were compared with that of the wild type. A total of 1499 ± 115 adherent bacteria were obtained per field of view in the control group, for only 322 ± 21 and 108 ± 7 in the *rstA‐* and *rstB*‐RNAi groups, respectively (Figure 3B). This finding indicated that the adhesion capability of *V. alginolyticus* was remarkably reduced after *rstA* and *rstB* silencing, respectively.

### 3.4 Effects of *rstA* and *rstB* silencing on motility

The motility abilities of *rstA*‐ and *rstB*‐silenced clones were also analyzed. As shown in Figure 3C, motility in *rstA‐* and *rstB*‐RNAi cells was significantly reduced, by 3.50‐ and 7.00‐fold, respectively.

### 3.5 Effects of *rstA* and *rstB* silencing on biofilm production

The biofilm formation ability of *V. alginolyticus* was remarkably reduced after *rstA* and *rstB* silencing compared with the control group, by 3.44‐ and 7.17 ‐fold, respectively (Figure 3D).

### 3.6 Effects of *rstA* and *rstB* silencing on hemolytic activity

Hemolytic activities were remarkably impaired in the *rstA‐* and *rstB*‐RNAi groups compared with the control group; *rstB*‐RNAi displayed a stronger suppression of hemolysis than *rstA*‐RNAi (Figure 3E).

### 3.7 Effects of *rstA* and *rstB* silencing on virulence

After artificial infection, mortality was dramatically lower in fish administered *rstA‐* and *rstB*‐RNAi strains, respectively, compared with the control group (Figure 3F). Survival rates were 0%, 65%, and 80% in groups infected with wild‐type, *rstA‐* RNAi and *rstB*‐RNAi strains, respectively. In the wild‐type, *rstA‐*RNAi, and *rstB*‐RNAi groups, death occurred at days 2, 3 and 4, respectively.

## 4 DISCUSSION

This study showed that *rstA* and *rstB* are involved in the regulation of adhesion, consistent with RNA‐Seq data. These findings supported the hypothesis that *rstA* and *rstB* sensitivity to environmental stresses might constitute a mechanism by which environmental conditions affect adhesion. Meanwhile, decreased motility was observed in *rstA‐* and *rstB*‐RNAi strains, indicating that *rstA* and *rstB* might influence adhesion by controlling motility. This is consistent with previous findings that RstB‐RstA system is necessary for motility in *Salmonella* (Tran et al., 2016).

Environmental factors can markedly influence the bacterial adhesion capacity. Indeed, pH, as an important environmental factor, significantly affects bacterial adhesion (Balebona et al., 1995; Yan et al., 2007). Heavy metals occurring in the environment also affect microorganisms (Haferburg & Kothe, 2007; Xiao, Zong, & Lu, 2015). Kong et al. (2015) reported that Cu^2+^, Pb^2+^, and Hg^2+^ significantly reduce *V. alginolyticus* adhesion to the skin mucus of large yellow croakers. However, the mechanisms underlying the effects of environmental factors on bacterial adhesion remain unclear. In this study, the effects of temperature, pH, and starvation on *rstA* and *rstB* expression levels were assessed.

The adhesion capability *V. alginolyticus* at various temperatures showed an inverted U‐shaped trend (Huang, Hu, et al., 2016; Huang, Huang, et al., 2016). *V. alginolyticus* showed remarkably stronger adhesion at 28°C compared with the other temperatures, which is consistent with the high frequency of vibriosis caused by *V. alginolyticus* in early summer (Baker‐Austin, Stockley, Rangdale, & Martinez‐Urtaza, 2010; Reilly, Reilly, Smith, & Baker‐Austin, 2011; Sterk, Schets, de Roda Husman, de Nijs, & Schijven, 2015). The trends of *rstA* and *rstB* gene expression levels and in vitro adhesion at various temperatures were similar; indicating that *V. alginolyticus* attachment was affected by temperature, with *rstA* and *rstB* likely contributing to adhesion control at different temperatures.

*V. alginolyticus* adhesion under diverse pH values also displayed an inverted U‐shaped trend, peaking at pH 7.0 (Huang, Hu, et al., 2016; Huang, Huang, et al., 2016). The trends of *rstA* and *rstB* gene expression levels and in vitro adhesion under diverse pH levels were similar, suggesting that pH affects *V. alginolyticus* adhesion, with *rstA* and *rstB* likely involved in adhesion control at different pH levels.

Huang, Hu, et al. (2016) and Huang, Huang, et al. (2016) demonstrated that starvation reduces *V. alginolyticus* adhesion, in a time‐dependent manner. The trends of *rstA* and *rstB* gene expression levels, and in vitro adhesion under starvation were very similar, suggesting that starvation affects *V. alginolyticus* attachment, with the involvement of *rstA* and *rstB*.

Taken together, these findings indicate that *rstA* and *rstB* play important roles in *V. alginolyticus* adhesion and are sensitive to certain environmental factors.

Biofilm production is a way by which bacteria protect themselves from the host immune system (She et al., 2016). However, whether *rstA* and *rstB* take part in biofilm formation remains undefined. The above results revealed that *rstA* and *rstB* silencing remarkably decreased biofilm formation. These findings indicated that *rstA* and *rstB* play key roles in *V. alginolyticus* biofilm formation.

Hemolysin is a chief virulence factor of many Vibrio (Syed et al., 2009). Terceti et al. (2017) found that *rstB* controls the production of damselysin, phobalysin C, and phobalysin P in *Photobacterium damselae* subsp. *damselae*. However, whether *rstA* and *rstB* regulate the hemolytic activity of *V. alginolyticus* remains unclear. In the present study, *rstA* and *rstB* silencing, respectively, in *V. alginolyticus* resulted in remarkably decreased hemolytic capability.

Several studies demonstrated that *rstA* and *rstB* are closely associated with bacterial virulence (Menanteau‐Ledouble & Lawrence, 2013). For example, *rstA* and *rstB* are involved in the regulation of invasion genes in *Salmonella enterica* Typhimurium (Menanteau‐Ledouble & Lawrence, 2013). The critical role of *rstB* in *P. damselae* subsp. *damselae* virulence for fish was also reported (Terceti et al., 2017). In the present study, the effects of *rstA* and *rstB* on *V. alginolyticus* virulence were also demonstrated. Monitoring fish postchallenge revealed that mortality was remarkably lower in groups treated with *rstA‐* and *rstB*‐RNAi strains compared with the control group. Meanwhile, death was delayed in animals injected with *rstA‐*RNAi and *rstB*‐RNAi strains compared with controls. These findings revealed that *rstA* and *rstB* knockdown has remarkable effects on *V. alginolyticus* virulence.

In conclusion, these findings suggest that: (1) *rstA* and *rstB* are tightly associated with *V. alginolyticus* adhesion; (2) *rstA* and *rstB* contribute to motility, biofilm production, hemolysis, and virulence in *V. alginolyticus*; (3) *rstA* and *rstB* modulate adhesion in response to environmental changes of temperature, pH, and starvation.

## CONFLICT OF INTEREST

None declared.

## References

[mbo3599-bib-0001] Baker‐Austin, C. , Stockley, L. , Rangdale, R. , & Martinez‐Urtaza, J. (2010). Environmental occurrence and clinical impact of *Vibrio vulnificus* and *Vibrio parahaemolyticus*: A European perspective. Environmental Microbiology Reports, 2(1), 7–18. 10.1111/j.1758-2229.2009.00096.x 23765993

[mbo3599-bib-0002] Balebona, M. C. , Morinigo, M. A. , Faris, A. , Krovacek, K. , Mansson, I. , Bordas, M. A. , & Borrego, J. J. (1995). Influence of salinity and pH on the adhesion of pathogenic *Vibrio* strains to *Sparus aurata* skin mucus. Aquaculture, 132, 113–120. 10.1016/0044-8486(94)00376-Y

[mbo3599-bib-0003] Benhamed, S. , Guardiola, F. A. , Mars, M. , & Esteban, M. Á. (2014). Pathogen bacteria adhesion to skin mucus of fishes. Veterinary Microbiology, 171, 1–12. 10.1016/j.vetmic.2014.03.008 24709124

[mbo3599-bib-0004] Bergstrom, K. , Kissoon‐Singh, V. , Gibson, D. L. , Ma, C. , Montero, M. , Sham, H. P. , … Chadee, K. (2010). Muc2 protects against lethal infectious colitis by disassociating pathogenic and commensal bacteria from the colonic mucosa. PLoS Pathogens, 6, e1000902. 20485566 10.1371/journal.ppat.1000902 PMC2869315

[mbo3599-bib-0005] Bradford, M. M. (1976). A rapid and sensitive method for the quantitation of microgram quantities of protein utilizing the principle of protein‐dye binding. Analytical Biochemistry, 72(1–2), 248–254. 10.1016/0003-2697(76)90527-3 942051

[mbo3599-bib-0006] Guo, L. , Huang, L. , Su, Y. , Qin, Y. , Zhao, L. , & Yan, Q. (2017). *secA*,* secD*,* secF*,* yajC*, and *yidC* contribute to the adhesion regulation of *Vibrio alginolyticus* . MicrobiologyOpen. 10.1002/mbo3.551 PMC5911994 29057613

[mbo3599-bib-0007] Haferburg, G. , & Kothe, E. (2007). Microbes and metals: Interactions in the environment. Journal of Basic Microbiology, 47(6), 453–467. 10.1002/(ISSN)1521-4028 18072246

[mbo3599-bib-0008] Huang, L. , Hu, J. , Su, Y. , Qin, Y. , Kong, W. , Ma, Y. , … Yan, Q. (2015). Identification and characterization of three *Vibrio alginolyticus* non‐coding RNAs involved in adhesion, chemotaxis, and motility processes. Frontiers in Cellular and Infection Microbiology, 5, 56. 26217589 10.3389/fcimb.2015.00056 PMC4498440

[mbo3599-bib-0009] Huang, L. , Hu, J. , Su, Y. , Qin, Y. , Kong, W. , Zhao, L. , … Yan, Q. (2016). Genome‐wide detection of predicted non‐coding RNAs related to the adhesion process in *Vibrio alginolyticus* using high‐throughput sequencing. Frontiers in Microbiology, 7, 619. 27199948 10.3389/fmicb.2016.00619 PMC4848308

[mbo3599-bib-0010] Huang, L. , Huang, L. , Yan, Q. , Qin, Y. , Ma, Y. , Lin, M. , … Zheng, J. (2016). The TCA pathway is an important player in the regulatory network governing *Vibrio alginolyticus* adhesion under adversity. Frontiers in Microbiology, 7, 40. 26870007 10.3389/fmicb.2016.00040 PMC4735382

[mbo3599-bib-0011] Huang, L. , Wang, L. , Lin, X. , Su, Y. , Qin, Y. , Kong, W. , … Yan, Q. (2017). *mcp*,* aer*,* cheB*, and *cheV* contribute to the regulation of *Vibrio alginolyticus* (ND‐01) adhesion under gradients of environmental factors. MicrobiologyOpen, 6(6). 10.1002/mbo3.517 PMC5727358 28744982

[mbo3599-bib-0012] Jiang, Q. , Chen, W. , Qin, Y. , Huang, L. , Xu, X. , Zhao, L. , & Yan, Q. (2017). AcuC, a histone deacetylase, contributes to the pathogenicity of *Aeromonas hydrophila* . Microbiologyopen, 6(4). 10.1002/mbo3.468 PMC5552924 28371510

[mbo3599-bib-0013] Jørgensen, M. G. , Nielsen, J. S. , Boysen, A. , Franch, T. , Møller‐Jensen, J. , Valentin‐Hansen, P. (2012). Small regulatory RNAs control the multi‐cellular adhesive lifestyle of *Escherichia coli* . Molecular Microbiology, 84, 36–50. 10.1111/j.1365-2958.2012.07976.x 22250746

[mbo3599-bib-0014] Kong, W. , Huang, L. , Su, Y. , Qin, Y. , Ma, Y. , Xu, X. , … Yan, Q. (2015). Investigation of possible molecular mechanisms underlying the regulation of adhesion in *Vibrio alginolyticus* with comparative transcriptome analysis. Antonie van Leeuwenhoek, 107(5), 1197–1206. 10.1007/s10482-015-0411-9 25726081 PMC4387256

[mbo3599-bib-0015] Lin, G. , Chen, W. , Su, Y. , Qin, Y. , Huang, L. , & Yan, Q. (2017). Ribose operon repressor (RbsR) contributes to the adhesion of *Aeromonas hydrophila* to *Anguilla japonica* mucus. MicrobiologyOpen, 6(4). 10.1002/mbo3.451 PMC5552941 28127946

[mbo3599-bib-0016] Liu, W. , Huang, L. , Su, Y. , Qin, Y. , Zhao, L. , & Yan, Q. (2017). Contributions of the oligopeptide permeases in multistep of *Vibrio alginolyticus* pathogenesis. MicrobiologyOpen, 6(5). 10.1002/mbo3.511 PMC5635161 28714216

[mbo3599-bib-0017] Liu, W. , Ren, P. , He, S. , Xu, L. , Yang, Y. , Gu, Z. , & Zhou, Z. (2013). Comparison of adhesive gut bacteria composition, immunity, and disease resistance in juvenile hybrid tilapia fed two different *Lactobacillus* strains. Fish & Shellfish Immunology, 35(1), 54–62. 10.1016/j.fsi.2013.04.010 23608032

[mbo3599-bib-0018] Luo, G. , Huang, L. , Su, Y. , Qin, Y. , Xu, X. , Zhao, L. , & Yan, Q. (2016). flrA, flrB and flrC regulate adhesion by controlling the expression of critical virulence genes in *Vibrio alginolyticus* . Emerging Microbes & Infections, 5(8), e85 10.1038/emi.2016.82 27485498 PMC5034100

[mbo3599-bib-0019] Menanteau‐Ledouble, S. , & Lawrence, M. L. (2013). Use of bioluminescence mutant screening for identification of *Edwardsiella ictaluri* genes involved in channel catfish (*Ictalurus punctatus*) skin colonization. Veterinary Microbiology, 162(2), 724–730. 10.1016/j.vetmic.2012.09.024 23092811

[mbo3599-bib-0020] Pan, X. , Yang, Y. , & Zhang, J. R. (2014). Molecular basis of host specificity in human pathogenic bacteria. Emerging Microbes & Infections, 3, e23 10.1038/emi.2014.23 26038515 PMC3974339

[mbo3599-bib-0021] Pfaffl, M. W. , Horgan, G. W. , & Dempfle, L. (2002). Relative expression software tool (REST) for group‐wise comparison and statistical analysis of relative expression results in real‐time PCR. Nucleic Acids Research, 30, e36 10.1093/nar/30.9.e36 11972351 PMC113859

[mbo3599-bib-0022] Pizarro‐Cerda, J. , & Cossart, P. (2006). Bacterial adhesion and entry into host cells. Cell, 124, 715–727. 10.1016/j.cell.2006.02.012 16497583

[mbo3599-bib-0023] Qiang, C. , Qingpi, Y. , Shen, M. , Zhixia, Z. H. , & Xiaoru, W. A. (2007). Adhesion of pathogenic *Vibrio alginolyticus* to the gill mucus of *Pseudosciaena crocea* . ATCA Oceanologica Sinica, 26(3), 101–109.

[mbo3599-bib-0024] Qin, Y. , Lin, G. , Chen, W. , Huang, B. , Huang, W. , & Yan, Q. (2014). Flagellar motility contributes to the invasion and survival of *Aeromonas hydrophila* in *Anguilla japonica* macrophages. Fish & Shellfish Immunology, 39(2), 273–279. 10.1016/j.fsi.2014.05.016 24859591

[mbo3599-bib-0025] Reilly, G. D. , Reilly, C. A. , Smith, E. G. , & Baker‐Austin, C. (2011). *Vibrio alginolyticus*‐associated wound infection acquired in British waters, Guernsey, July 2011. Eurosurveillance Weekly, 16(42), 10. 22027377

[mbo3599-bib-0026] She, P. , Chen, L. , Qi, Y. , Xu, H. , Liu, Y. , Wang, Y. , … Wu, Y. (2016). Effects of human serum and apo‐Transferrin on *Staphylococcus epidermidis* RP62A biofilm formation. Microbiologyopen, 5(6), 957–966. 10.1002/mbo3.379 27185376 PMC5221445

[mbo3599-bib-0027] Sterk, A. , Schets, F. M. , de Roda Husman, A. M. , de Nijs, T. , & Schijven, J. F. (2015). Effect of climate change on the concentration and associated risks of *Vibrio Spp*. Dutch recreational waters. Risk Analysis, 35(9), 1717–1729. 10.1111/risa.12365 25809307

[mbo3599-bib-0028] Syed, K. A. , Beyhan, S. , Correa, N. , Queen, J. , Liu, J. , Peng, F. , … Klose, K. E. (2009). The *Vibrio cholerae* flagellar regulatory hierarchy controls expression of virulence factors. Journal of Bacteriology, 191(21), 6555–6570. 10.1128/JB.00949-09 19717600 PMC2795290

[mbo3599-bib-0029] Taghavi, S. , Wu, X. , Ouyang, L. , Zhang, Y.B. , Stadler, A. , McCorkle, S. , … Van der Lelie, D. (2015). Transcriptional responses to sucrose mimic the plant‐associated life style of the plant growth promoting endophyte *Enterobacter* sp. 638. PLoS ONE, 10(1), e0115455 10.1371/journal.pone.0115455 25607953 PMC4301647

[mbo3599-bib-0030] Terceti, M. S. , Rivas, A. J. , Alvarez, L. , Noia, M. , Cava, F. , & Osorio, C. R. (2017). rstB regulates expression of the *Photobacterium damselae* subsp. damselae major virulence factors Damselysin, Phobalysin P and Phobalysin C. Frontiers in Microbiology, 8, 582. 28443076 10.3389/fmicb.2017.00582 PMC5385354

[mbo3599-bib-0031] Tran, T. K. , Han, Q. Q. , Shi, Y. , & Guo, L. (2016). A comparative proteomic analysis of *Salmonella typhimurium* under the regulation of the RstA/RstB and PhoP/PhoQ systems. Biochimica et Biophysica Acta (BBA)‐Proteins and Proteomics, 1864(12), 1686–1695. 10.1016/j.bbapap.2016.09.003 27618760

[mbo3599-bib-0032] Tsou, A. M. , & Zhu, J. (2010). Quorum sensing negatively regulates hemolysin transcriptionally and posttranslationally in *Vibrio cholerae* . Infection and Immunity, 78, 461–467. 10.1128/IAI.00590-09 19858311 PMC2798175

[mbo3599-bib-0033] Wang, Y. D. , Huang, S. J. , Chou, H. N. , Liao, W. L. , Gong, H. Y. , & Chen, J. Y. (2014). Transcriptome analysis of the effect of *Vibrio alginolyticus* infection on the innate immunity‐related complement pathway in *Epinephelus coioides* . BMC Genomics, 15, 1102 10.1186/1471-2164-15-1102 25496447 PMC4407539

[mbo3599-bib-0034] Wang, L. , Huang, L. , Su, Y. , Qin, Y. , Kong, W. , Ma, Y. , … Yan, Q. (2015). Involvement of the flagellar assembly pathway in *Vibrio alginolyticus* adhesion under environmental stresses. Frontiers in Cellular and Infection Microbiology, 5, 59. 26322276 10.3389/fcimb.2015.00059 PMC4533019

[mbo3599-bib-0035] Wu, C. , Zhang, D. , Kan, M. , Lv, Z. , Zhu, A. , Su, Y. , … Jiang, L. (2014). The draft genome of the large yellow croaker reveals well‐developed innate immunity. Nature Communications, 5, 5227 10.1038/ncomms6227 PMC4263168 25407894

[mbo3599-bib-0036] Xiao, Q. , Zong, Y. , & Lu, S. (2015). Assessment of heavy metal pollution and human health risk in urban soils of steel industrial city (Anshan), Liaoning, Northeast China. Ecotoxicology and Environmental Safety, 120, 377–385. 26114257 10.1016/j.ecoenv.2015.06.019

[mbo3599-bib-0037] Yamamoto, K. , Hirao, K. , Oshima, T. , Aiba, H. , Utsumi, R. , & Ishihama, A. (2005). Functional characterization in vitro of all two‐component signal transduction systems from *Escherichia coli* . Journal of Biological Chemistry, 280(2), 1448–1456. 10.1074/jbc.M410104200 15522865

[mbo3599-bib-0038] Yan, Q. , Chen, Q. , Ma, S. , Zhuang, Z. , & Wang, X. (2007). Characteristics of adherence of pathogenic *Vibrio alginolyticus* to the intestinal mucus of large yellow croaker (*Pseudosciaena crocea*). Aquaculture, 269(1), 21–30. 10.1016/j.aquaculture.2007.02.042

